# Maternal, neonatal, and child health systems under rapid urbanization: a qualitative study in a suburban district in Vietnam

**DOI:** 10.1186/s12913-019-4874-7

**Published:** 2020-02-05

**Authors:** Jongho Heo, Seung Yun Kim, Jinseon Yi, Soo-Young Yu, Da Eun Jung, Sangmi Lee, Ju Youn Jung, Hyunsuk Kim, Ngan Do, Hwa-Young Lee, You-Seon Nam, Van Minh Hoang, Ngoc Hoat Luu, Jong-Koo Lee, Thi Giang Huong Tran, Juhwan Oh

**Affiliations:** 1 National Assembly Futures Institute, Seoul, Republic of Korea; 2 JW LEE Center for Global Medicine, Seoul National University College of Medicine, Seoul, Republic of Korea; 3 UNICEF Kenya Country Office, United Nations Children’s Fund, Nairobi, Kenya; 4 College of Nursing, Seoul National University, Seoul, Republic of Korea; 5 Medipeace, Seoul, Republic of Korea; 6 Korea International Cooperation Agency, Seongnam-si, Republic of Korea; 7 Department of Biomedical Science, Seoul National University College of Medicine and Hospital, Seoul, Republic of Korea; 8 Department of Internal Medicine, College of Medicine, Hallym University, Chuncheon-si, Republic of Korea; 9 Sphere Institute, Burlingame, CA USA; 10 Takemi Program in International Health, Department of Global Health and Population, Harvard T.H. Chan School of Public Health, Boston, MA USA; 11 Department of Family Medicine, Seoul National University College of Medicine, Seoul, Republic of Korea; 12 Hanoi University of Public Health, Hanoi, Vietnam; 13 Hanoi Medical University, Hanoi, Vietnam; 14 Department of Social and Behavioral Sciences, Harvard T.H. Chan School of Public Health, 677 Huntington Ave, Boston, USA

**Keywords:** Access, MNCH, Quality of service, Urbanization, Antenatal care, Delivery

## Abstract

**Abstract:**

**Background:**

Vietnam has been successful in increasing access to maternal, neonatal, and child health (MNCH) services during last decades; however, little is known about whether the primary MNCH service utilization has been properly utilized under the recent rapid urbanization. We aimed to examine current MNCH service utilization patterns at a district level.

**Methods:**

The study was conducted qualitatively in a rural district named Quốc Oai. Women who gave a birth within a year and medical staff at various levels participated through 43 individual in-depth interviews and 3 focus group interviews.

**Results:**

Primary MNCH services were underutilized due to a failure to meet increased quality needs. Most of the mothers preferred private clinics for antenatal care and the district hospital for delivery due to the better service quality of these facilities compared to that of the commune health stations (CHSs). Mothers had few sociocultural barriers to acquiring service information or utilizing services based on their improved standard of living. A financial burden for some services, including caesarian section, still existed for uninsured mothers, while their insured counterparts had relatively few difficulties.

**Conclusions:**

For the improved macro-efficiency of MNCH systems, the government needs to rearrange human resources and/or merge some CHSs to achieve economies of scale and align with service volume distribution across the different levels.

## Background

By virtue of the UN Millennium Development Goals (MDGs), the maternal and under five mortality rates were reduced from 1990 to 2015 at all levels of national income [1]. During the MDG era, Vietnam was one of the most countries that showed remarkable progress in maternal, neonatal, child health (MNCH). It showed a successful decrease in the estimated infant mortality rate, from 36.6 in 1990 to 17.3 in 2015 [2]. The percentage of Vietnamese mothers who have four or more antenatal care (ANC) visits has increased from 29% in 2002 to more than 70% in 2014 [3]. In addition, from 1993 to 2011, over 90% of the children under age 5 received the immunizations recommended by the National Expanded Program on Immunization [4].

This result was attributable to the Vietnam government’s efforts to improve the access to and the quality of maternal services at commune health stations (CHSs), emphasizing the quality of primary care facilities, procurement of medical equipment, and training for health care providers [5]. Vietnam’s national strategy on reproductive health care service [6] recommends that CHSs need to recognize the high-risk factors and complications through at least three ANC visits for uncomplicated pregnancies. CHSs perform normal delivery and the supervision of or assistance with home delivery. In cases of the early recognition of labor complications such as obstetric hemorrhage or newborn problems such as asphyxia, CHSs are to refer patients to upper-level medical services. For postnatal care (PNC), CHSs assist with immunizations as scheduled and encourage breastfeeding. District hospitals (DHs) perform caesarian sections (C-sections), manage high-risk pregnancies, care for low birth weight and premature babies and provide all services at the CHS level. Private clinics (PCs) provide primary MNCH services to supplement the public sector services and improve the efficiency of the public sector after the “Doi Moi” policy introduced user fees and private health sectors in 1986 [7].

Based on the remarkable progress in the MDG era, the Ministry of Health announced a 5-year health sector plan in 2011–2015 to consolidate the health care delivery structure from the central to the local level, especially the grass-roots health network, to continue strengthening the health care delivery system. Additionally, the Vietnam government is planning to increase the yearly state budget for health, striving to allocate at least 10% of the state budget to health to cover investments and recurring costs [5].

Meanwhile, Vietnam has been challenged to maintain a sustainable health care system mainly due to its rapid economic growth and urbanization. Traditionally, Vietnamese society has been strongly influenced both by the communism and Confucian values; however, Vietnam has been undergoing fundamental changes towards a market-oriented society since the late 1980s [7]. As a result, the Vietnamese economy showed some of the most rapid and largest growth in the world in the last decade. Parallel to socioeconomic development, 29.6% of the total population lived in urban areas, compared to 23.7% in 1999 [5]. Based on the improved standard of living, health care needs have rapidly increased, and help-seeking behaviors for health services have also diverged, especially among those who live in urban areas. These rapid socioeconomic transitions also have created challenges in MNCH with respect to the quality of services and the appropriate combination of service provisions. For example, urbanization leads to an increase in urban births, creating additional challenges in the capacity for MNCH services to improve the access to and quality of obstetric care [8].

However, little is known about whether the primary MNCH service utilization in CHSs has been appropriate in terms of macro-efficiency under the recent rapid urbanization. Although there have been a few qualitative studies on MNCH service utilizations and related health behaviors in Vietnam, comprehensive qualitative studies on the status quo of primary MNCH service utilization in urbanizing contexts have been rare. Considering that access to health services is a complex and continuous process that involves changes in the social values, economic interests, and political processes of a society [9, 10], adjusting health systems to respond to societal changes may be recommended for proper MNCH service utilization, better macro-efficiency and the eventual sustainability of health systems.

In this qualitative descriptive study, we explored access to MNCH services through interviews of the supply and demand sides of MNCH services in a suburban area of Hanoi, where urbanization has been actively ongoing. Based on a comprehensive framework for access, we aim to (1) identify determinants in primary MNCH service utilization by exploring the status quo of MNCH service utilization with a theoretical framework on service access and (2) suggest policy implications for better macro-efficiency of MNCH systems.

## Methods

### Study area and population

The subjects of this study were health care providers and service users living in the Quốc Oai district in the Red River Delta region of Vietnam. The Quốc Oai district is located 30 km away from western Hanoi, with an area of 147km^2^ and a population of 163,355 people in 2009. The district was once a rural area but is now experiencing rapid urbanization. This district is included in the Hanoi region, and the Kinh people account for 99% of this region’s ethnicity.

Convenience sampling was used to recruit participants for both the user and provider sides of the study. The whole recruitment process was discussed with and conducted by CHSs via telephone. For demand-side interviews, 30 women (aged 21–33) who gave a birth within a year and their family member were selected among those enrolled in CHSs. To complement the representativeness of the sample, participants’ distance from CHSs and the income level (high/middle/low) were considered in the sampling. For provider-side interviews, 10 medical providers from CHSs (*N* = 6), the district hospital (DH) (*N* = 2), the district health center (*N* = 1), and the district health office (*N* = 1) participated in the study. None of the invited individuals refused to participate or withdraw consent. All participants signed written consent forms. In total, 40 individual interviews (IDIs) and three focus group interviews (FGIs) were conducted. Two FGIs were performed with users and one with providers with four to seven participants in each group. The general information of the participants is presented in Table 1.

**Table 1:** Information of the participants

Interview number	Identity	Location	User/Provider
IDI 1~29	Mother	Commune	User
IDI 30	Grandmother		
IDI 31~36	2 Vice Heads of CHS	CHS	Provider
	Manager of CHS		
	2 Midwives		
	Health worker		
IDI 37~38	Vice director of DH	DH	Provider
	Obstetrician		
IDI 39	Director of DHC	DHC	Provider
IDI 40	Head of DHO	DHO	Provider
FGI 1	Head of obstetric division	DH	Provider
	3 Midwives		
FGI 2	4 Grand mothers	Sai Son	Senior
	1 Grand father	Commune	
FGI 3	5 Mothers	Yen Son	User
		Commune	

*IDI* In-depth interview, *FGI* Focus group interview, *CHS* Commune Health Station, *DH* District hospital, *DHC* District Health Center, *DHO* District Health Office

### Interview process

Interviews for data collection were conducted from July 31 to August 4, 2016, in the Quốc Oai district of Hanoi. Structured open-ended questions were used through the interview process. A question guide was constructed in advance. For user interviews, questions were composed of general information, health-seeking behaviors, maternal and child health, and immunization information. For providers, there was information about health care delivery systems, the kinds of services provided in health facilities, equipment, resource supply, and major MNCH issues in the district and the effects of its related policies, workloads and working conditions. Following open-ended questions, probes were used to explore participants’ opinions on access to health care services in the Quốc Oai district. For the interview guides for users and providers, please see Additional file 1. To establish relationships with participants, interviews were initiated with the introduction of the research teams and a talk with their newborns so that participants felt that they were important to the interviews, followed by a short introduction of the research. All interviews were conducted by three interview teams, consisting of one leader and three to four members of each team. Team leaders (JH, HL, and JO), who have Ph.D. degree in public health, moderated FGIs and led IDIs. Team members (SYK, JY, SYY, NDK, HL, and YSN: medical doctors, registered nurses, and doctoral students in public health) supported the interviews through obtaining written consent forms, recording interviews, making field notes to capture important elements, providing compensation, and making other arrangements. The interview teams were trained in degree coursework or various programs regarding qualitative study methods. The IDIs and FGIs lasted approximately 1.5~2 h until the information from an interviewee reached saturation (no new or relevant information emerged). During the interviews, the moderator and interviewers had a neutral and intersubjective position. JH and JO were male interviewers.

Every interview team had a Vietnamese interpreter who translated Vietnamese to English and English to Vietnamese during the interviews. Every team member was fluent in English or Vietnamese. All dialogues were recorded and later transcribed into English. The IDIs were conducted at the Quốc Oai town commune hall, CHSs, and interviewees’ houses located in the Liep Tuyet commune and the Cong Hoa commune. The FGIs also took place in the Quốc Oai town commune hall and a conference room at the DH. During the data collection period, coauthors held a conference every night to check the data saturation status. A peer debriefing process was involved in the conference, which ensures the credibility of the research by reducing the bias of a single researcher. Since no new information emerged in 3rd FGI and the 43rd in-depth interview (IDI), it was concluded that the data were saturated.

### Interview analysis

A direct content analysis method was applied to analyze data [11]. Unlike conventional content analysis, it is a more structured and deductive process that uses existing theory or a conceptual framework [12].

**Fig. 1:**
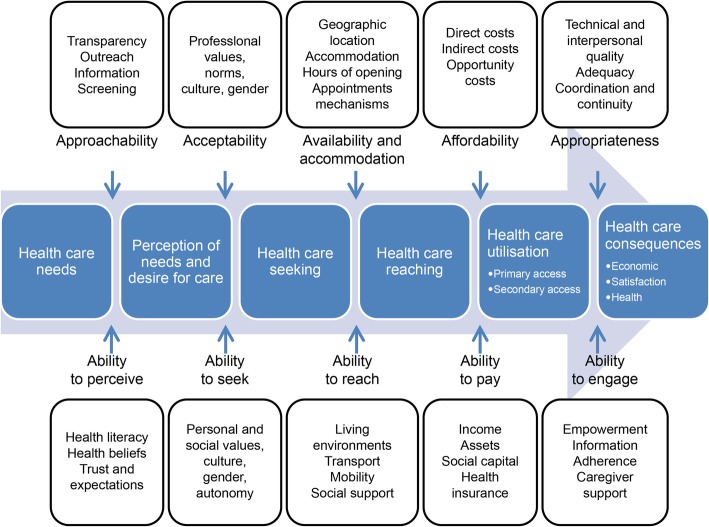
A conceptual framework for access to health care (From Levesque et al. (2013), reproduction of the figure is permitted)

We employed the health care access framework suggested by Levesque et al. [9], highlighted by a comprehensively broad dimension and determinants that integrate the demand and supply side all along the process of service utilization (Fig. 1). In this framework, there are five dimensions of accessibility conceptualized on the side of health systems, institutions, organizations, and providers: P (provider-side)-1) approachability; P-2) acceptability; P-3) availability and accommodation; P-4) affordability; and P-5) appropriateness. Individuals, households, communities, and populations interact with the dimensions of accessibility to create access. These five dimensions of abilities are: U (user-side)-1) ability to perceive; U-2) ability to seek; U-3) ability to reach, U-4) ability to pay; and U-5) ability to engage. Each of the definitions is summarized in Table 2. These dimensions capture supply- and demand-side determinants; for example, direct costs for affordability match health insurance for the ability to pay. More detailed information on the theoretical framework can be found elsewhere [9].

**Table 2:** Definition of each dimension in access to health care

Division	Dimension	Definition
Provider side	Approachability	“It is the ability when facing health needs to identify some form of services exists, can be reached, and have an important on the health of individuals.”
	Acceptability	“It is the possibility for people to accept the aspects of the service in cultural and social context and the judged appropriateness for the person to seek care.”
	Availability and accommodation	“Health services can be reached both physically and in a timely manner. It results from health facilities, urban contexts, individuals, characteristics of providers, modes of provision of services and so on.”
	Affordability	“It is the economic capacity to consume resources and time for people to utilize appropriate services.”
	Appropriateness	“It is the adequacy of service and client’s needs, its timeliness, and the expense of care in assessing health problems and deciding the appropriate treatment and technical and interpersonal quality of the health care services.”
User side	Ability to perceive	“Perceiving needs for care is important and it can be determined by factors such as health literacy, knowledge about health and beliefs related to health and sickness.”
	Ability to seek	“It is related to the concept of personal autonomy and capacity to choose the seek care, knowledge about health care options and individual rights that would determine to express the intention to obtain health care.”
	Ability to reach	“It is the personal mobility and availability of transportation, occupational flexibility, and knowledge about health services which would enable one person to physically reach service providers.”
	Ability to pay	“It is the ability to create economic resources to provide health care services without catastrophic expenditure required for basic necessities.”
	Ability to engage	“It is client involvement and participation in decision-making and treatment decisions, which is strongly determined by the ability and motivation to participate in care and commit to its completion.”

Note: All the definitions were from Levesque et al. (2013)

We adopted this framework to explore how primary MNCH service utilization has been appropriate. If the service has not been properly utilized, we identified determinants of the inadequacy within the framework. In particular, we explored supply-side determinants across three levels (CHS, DH, and PC) of the MNCH system within the study district, as the inadequacy of primary MNCH service utilization may be caused by systematic problems, including unnecessary competition or a lack of coordination across levels of service providers.

Initial codes were directly derived from the interview texts and sorted into dimensions and determinants of the theoretical framework using an Excel sheet (Microsoft Office 2011, Microsoft Cooperation, USA). All coding and sorting processes were double-checked by the coauthors. For a review of the quality of reporting, the COREQ (Consolidated Criteria for Reporting Qualitative Research) checklist was used.

## Results

### Approachability (P-1) and ability to perceive needs for care (U-1): information on MNCH services was well disseminated

Multiple organizations, including the district health center (DHC), the District Population Center (DPC), the DH, and CHSs, disseminated information on MNCH services through various activities, including community campaigns, the outreach efforts of health staff/midwives, educational programs, and the dissemination of instructional materials. The DHC organizes overall health education events to provide health knowledge directly and indirectly.

*“We have a department for health education. We normally organize events to give knowledge to a community on topics including ANC, HIV, food security, and so on. We call these efforts “direct health education.” For “indirect” health education, we make publications and put information on the radio.” (Director of DHC).*

Demographic coordinators monitored the conditions of pregnant women and children in each village. CHSs delivered invitation cards to pregnant women for their regular vaccination check-ups. On vaccination days, CHSs additionally conducted regular health campaigns for women about family planning and contraception methods.

*“In this campaign, women are supposed to come and have a screening. There are around 20 women on average in every campaign.” (Vice head of CHS).*

*“They (demographic coordinators) maintain vaccination notebooks on those who need to have which vaccinations. So normally, mothers bring their kids to a community health center to get vaccinations on the scheduled day noted in invitation cards.” (Mother).*

Mothers had high health literacy regarding MNCH. They were well aware of healthy foods, necessary nutrition, and medicine during pregnancy. All the mothers participating in our study recognized the importance of vaccination. They also knew where to visit for regular check-ups and how to handle their children’s illness.

*“During the pregnancy, I ate more fruit and vegetables and iron and calcium to improve the health of my body.” (Mother).*

### Acceptability (P-2) and ability to seek health care (U-2): autonomously exploring health services

In the suburban contexts, there were few cultural or social barriers to accepting the aspects of MNCH services at all levels. Most obstetric doctors were male; however, there was no cultural belief or societal forbiddance of physical contact between male staff and female patients.

All the women participating in our study also experience few cultural or social barriers to seeking MNCH services. The decision making of all the women who sought services was made based on their high level of autonomy and ability to seek proper services. Most pregnant women obtained information on MNCH services mainly from the internet, their families, friends, and from formal routes including medical staff or instructive materials. Mothers were able to discern differences in the type and quality of services among medical providers and decided where to visit based on the seriousness of an illness and the service quality of the providers. For a mild case, they preferred to use traditional medicine or visit a pharmacy. They believed that it was necessary to visit the DH in severe cases, as this facility can handle more complicated cases.

*“I found information by searching the internet and through magazines.” (Mother).*

*“When my baby has an illness like a cough, fever, or runny nose, I usually use traditional medicine. Itis very effective for these symptoms. Sometimes, I also buy some medicines from a pharmacy.” (Mother).*

*“I think that the community health center cannot determine exactly what happened to my child and me. For common illnesses, I can treat myself, but for more severe illnesses, the community health center is not qualified to determine exactly what happened.” (Mother).*

### Availability/accommodation (P-3) and ability to reach health care (U-3): preference for PCs and the DH due to their excellence in equipment, facilities, and manpower

Mothers in our interviews chose health facilities based on whether they provide the services in a physically and timely manner, including better facilities, devices, and flexibility of working hours. During pregnancy, women generally preferred to visit PCs, rather than the DH or CHSs, for several reasons: closer proximity to villages, open hours after work, sex identification, and the provision of colorful ultrasound images. For delivery, the majority of women used the DH, as it provided better services through more equipment and better facilities as well as more skillful medical personnel. CHSs were located relatively near where they lived; however, they offered basic-level delivery services in out-of-date commodities and facilities, which are insufficient to meet women’s expected quality of service. Some of the women planned to deliver a child in a CHS; however, they were referred to the DH, as they had complex cases, such as C-sections, that CHSs could not handle.

*“I wanted to know whether the baby was a boy or a girl. However, when I got an ultrasound in a CHS or even in the DH, staff didn’t say the child’s sex. However, the doctor in the PC let me know it.” (Mother)*

“*The private clinic is available until 9 PM, but the hospital is available until 4 PM. They also don’t work on the weekends.” (Mother)*

*“I had an ultrasound image at a private clinic one month, and I went to a CHS two times only for vaccinations for my babies.” (Women who delivered in the DH)*

*“A woman who needs C-section will be referred to the DH.” (Vice head of CHS)*

*“The staff of a CHS were helpful and very friendly, and the equipment was able to provide services. However, in terms of things to be improved, the equipment was slightly old.” (Mother).*

CHSs were preferred to other health providers only for babies’ vaccinations. Due to the logistics of vaccines, CHSs fixed one or 2 days (usually the 5th of every month) only for vaccinations for scheduled mothers and children. If mothers missed those days, they had to wait for the next immunization day. However, none of the mothers participating in the study missed the vaccination days. After administering injections, the CHS staff also monitored side effects, including fevers. Except for minor complaints about long waiting times on immunization days, most mothers were satisfied with the services. Some CHSs used a time slot reservation system for villages to reduce waiting time.

*“I think the immunization service is fine. There is no suggestion for improvement. (Mother).*

*“At my commune, the CHS allocates different time slots for different villages. That’s why I didn’t wait for so long.” (Mother).*

Most of the women did not complain about transportation, as the study commune was relatively close to the DH. Motorcycles were the most frequently used for the mode of transportation, in addition to taxis and walking.

*“I am not close to the hospital, so I go once every three months. However, if I lived closer to the facility, then I would go once a month.” (Mother).*

*“I lived in a village less than 1 km far from the DH. My relative took me to the DH by motorcycle.” (Women who delivered in the DH).*

### Affordability (P-4) and ability to pay for health care (U-4): the financial burden of cesarean section for the uninsured

Women needed to pay an average medical cost of about VND 100,000 (USD 4.4) only for color ultrasonography in PCs. However, they still preferred this option because the cost was affordable. ANC in the DH was not favored because of the long waiting time (approximately for 2~3 h).

*“Each time, I paid VND 100,000 for ultrasonography (in a PC). However, I feel that it is not too expensive. It is just normal.” (Mother).*

Delivery in the DH or CHSs was free of charge with health insurance; however, delivery could be an overly burdensome if uninsured women needed C-sections. One of the women we interviewed inevitably decided to have a normal delivery even if she needed a C-section, since she knew that a C-section would be very expensive without insurance but had the chance to have a normal delivery (approximately VND 2 million (USD 88)).

*“My first delivery was quite hard because of the baby’s position. I truly wanted to have C-section, but the doctor said that I have the chance (to have normal delivery) and the cost of C-section is much more than a normal delivery. Since I didn’t have any health insurance, I waited to give a birth. Finally, I had the baby after 10 hours of suffering.” (Mother).*

Unofficial or ‘under the table payment’ may be another barrier that limited access to the DH. One of the interviewees who used the DH for delivery had to pay an unofficial VND 50,000 (USD 2.2) to the doctor as well as VND 2 million (USD 88) for delivery. It was not necessary for patients to offer bribes for every visit, but it was customary especially for hospitalization. If they did not, sometimes they experienced discrimination in the attitudes or services of hospital staff.

*“The attitude of the nurse in the district hospital was very rude. She shouted me for not giving (under the table) money. One of the large differences between commune health stations and the district hospital was the kindness of the staff.” (Mother).*

Health service utilization for children under age 6 was free of charge due to the government health insurance program. Essential vaccinations were also available on the vaccination days free of charge at the CHSs. However, mothers had to pay for vaccinations outside essential categories such as seasonal flu.

*“The vaccination was free because it comes from the National Program.” (Mother).*

*“My children under the age of 6 years have their health insurance. Therefore, the payment is free.” (Mother).*

### Appropriateness (P-5) and ability to engage in health care (U-5): the large quality gap between the DH and CHSs

For effective and efficient MNCH service delivery, the governmental health system stratified different levels of health service providers including the DH, CHSs, and PCs. Although there are differences in service quality across the levels, the services provided also overlapped substantially. The DH provides ANC, delivery, PNC, and complicated obstetric care for premature infants. CHSs also provide ANC, delivery, and PNC, but the services are so basic that they cannot meet all the needs of the mothers. However, PCs meet the women’s service needs through professional consultants. In many cases, a doctor in the DH had a dual position as a doctor in a PC. The majority of women bypassed CHSs, as PCs were preferred for ANC and the DH was preferred for delivery, which led to a lack of practical training for ANC and delivery in CHSs to maintain staff knowledge and skill. This also resulted in a low volume of MNCH service provision in CHS, which, in turn, led to inefficient standby and night shifts of midwives for rare cases of delivery and ANC; whereas, the work-load in the DH was excessive due to insufficiency of human resources, such as midwives. This, in turn, increased dissatisfaction among both service users and providers as well as increased the inefficiency of the health system.

*“No patients in the CHS. From the beginning of this year until July, there were only 8 cases. In addition, 55 pregnant women visited for a health check-up, and the time spent on the health check-up was as short as approximately 5 minutes.” (Director of a CHS).*

*“I have to work 8 hours a day, two night-shifts per week. Outside these hours, I also need to come to here when something happens.” (Midwife in a CHS).*

*“In the DH, 8 to 15 patients are hospitalized every day, and 2-3 times more patients visit for ANC. There are 3,500 deliveries, of which 40% were C-sections.” (Obstetrician in the DH).*

In terms of the ability to engage in health care, once a woman chose the health facilities she wanted to go, she generally complied with the instructions of the medical staff based on trust. Some of the women engaged actively in treatment decisions.

*“I just went to the DH when my child had severe illness. I wanted to get services such as CT scans or something like that.” (Mother).*

## Discussion

Our study aimed to identify determinants in primary MNCH service utilization by exploring the current status of the MNCH service utilization in the Quốc Oai district in Vietnam, where socioeconomic transition and urbanization have accelerated. The most prominent findings of this study were as follows: (1) primary MNCH services were underutilized due to a low level of perceived service quality compared to mothers’ expectations: most of the mothers preferred PCs for ANC and the DH for delivery due to their excellence in the availability and appropriateness of their services compared to those of CHSs; (2) based on their improved standard of living, mothers have few sociocultural barriers to acquiring MNCH related service information or utilizing MNCH services; and (3) although most of the mothers were insured, there still existed a financial burden for some MNCH services for the uninsured.

The findings in our study showed levels of access to MNCH services that differed from those depicted in previous studies. Past studies conducted in rural areas or a decade ago identified factors that limited access to MNCH services including women’s low decision-making abilities, economic constraints, cultural barriers, or geographic distances [13, 14]. Then, they mainly suggested quality improvement of CHSs to increase access to essential or facility-based MNCH services [13, 15]. However, in the current suburban setting in Vietnam, all the women had more than three ANC visits during their pregnancy; thus, meeting national criteria was no longer a problem in the district: rather, the quality of ANC services mattered [16]. The need for higher-quality ANC and delivery services resulted in crowding in the DH and PCs and bypassing CHSs. Only two studies captured the early stage of such changes in MNCH service utilization [17, 18]. The studies suggested quality improvement of CHSs after they found that many women were turning to PCs and public hospitals, especially in urban areas, due to the fact that CHSs were falling short in meeting patient expectations in terms of service quality. Our study showed that the need for better MNCH service quality has increased among mothers and now seems to be normative even in suburban areas.

Most of the mothers who participated in our study utilized PCs for ANC and the DH for delivery due to their better service quality than that of CHSs. In terms of availability, ANC in PCs was preferred mainly due to their open hours after work, better facilities, professional counseling including sex identification, and colorful ultrasonography. Fees for ANC in PCs were affordable for the mothers, given their improved standard of living. However, the needs among mothers were mostly not met by free ANC in CHSs, as they provided only black-and-white ultrasound images without any professional counseling. As women living in suburban areas participate in the workforce, including working in companies or factories, their needs for ANC after work may be increased. Notably, most of the women noted the colorful ultrasound images as an important reason for their preference for PCs for ANC even though it is not a core component of ANC in the national guidelines [16]. They may adopt modern health care services more easily and rapidly in the urban contexts [19]. This finding may be due to the escalating need for higher-quality ANC services among women and the commercialization of the Vietnamese health care system.

Regarding delivery services, a large portion of mothers preferred the DH as it provided better service quality than CHSs. In particular, better equipment and service quality may be major determinants, as the DH was relatively close to the study area and had been recently renovated. Some patients had to use the DH as they were referred from CHSs for C-sections or other emergencies. Midwives and doctors in CHSs were experienced in basic delivery; however, they were not allowed to handle more dangerous cases due to regulations. Thus, women in urban areas may prefer delivery in the DH to avoid possible emergency situations and to enjoy safer and cleaner facilities with more professional medical staff [20, 21]. Compared previous studies, there were no previous cases of delivery at home that had been conducted widely with trained birth attendants or private providers in Vietnam [13]. Previous studies showed that deliveries at home attended by health staff were thought to be convenient, affordable and safe [13]. However, in the current suburban setting, all the women regarded delivery in health facilities as normative. The government’s efforts to increase facility-based deliveries might have contributed. Furthermore, pregnant women may be able to access higher-quality delivery services in the DH owing to their improved socioeconomic status, better knowledge and understanding of childbirth, and reduced workload.

Another prominent finding was that mothers decided autonomously where to utilize the MNCH services based on their knowledge. They reported that they experienced few cultural, social, or religious factors that limited their ability to obtain health information or to access services. Previously, pregnant women in Vietnam were in a socially, economically, and culturally vulnerable position, especially under scarce family resources [13]. Traditional Confucian culture encouraged women to comply with the decision of her husband and parents-in-law [22, 23], and the religious beliefs of the family influenced decision making, especially in rural areas [13]. However, women we interviewed were independent in the decision on service utilization without any prohibition or interference from their husbands and mother-in-law during pregnancy and childbirth. There were also no women who had son preferences or pressure to have a son from family, which previous studies reported [23]. A rapid economic growth and social transition may lead women in the suburban area to have jobs other than farming, in turn, to have economic power and higher position in the family than before. Indeed, most of the women we interviewed had jobs such as working in a pharmacy, a store, and a company before they had a child birth. This change may also be partly attributed to the transition to small family structures in the urbanizing setting. A large portion of women lived only with a husband and children or in some cases with their parents to help childcare, so they were easier to make their own decision [15].

Third, we found that economic constraints on access to MNCH services persisted among some women even in the same setting. Most of the women were insured, but some were not. Health insurance for adults was usually bought from workplaces. Therefore, people without a formal job or self-employed had to sign up for health insurance separately, but some people were not able to get insured due to unaffordable premium. For this reason, one of the interviewees decided not to obtain a C-section even if it was needed. Although our study did not aim to examine the extent to which economic inequality expanded in the study area, the urban health literature concerned increasing inequality in access to health services especially when a society has undergone rapid sociodemographic changes and economic growth [20, 24]. Previous studies also noted that rapid but inequitable socioeconomic development in Vietnam had increased health disparities [25]. Especially among low-income groups, in addition to the formal fee and indirect costs such as transportation or time, bribe money contributed to a low utilization of MNCH services [26].

Finally, we found that immunization and child health service utilization were properly performed. Almost all the women we interviewed were very satisfied with the services. All the mothers participated in our study recognized the importance of vaccination and received their children vaccinated on the schedules. Health service utilization for children until 6 was free of charge due to the government health insurance program. We did not find any cases of bypassing CHSs in vaccination.

Our study has limitations that require caution in interpreting study results. First, our study was not able to identify ethnic disadvantages in MNCH service utilizations because all our samples were from the Kinh, the major ethnic group, which composed 99% of this district. As a previous study, minor ethnicity was the factor that hampered the ANC attendance of Vietnamese women [27]; further study may need to consider the difference in access to MNCH services in the urbanization. Second, our findings may not represent the situations in the district as a whole, as we used a convenience sampling method to recruit participants for the study even when we sampled considering the distance from CHSs and the income level.

## Conclusion

Based on the findings in our study, several alternative MNCH service provision reform options could be considered. The first policy option is a rearrangement of human resources to align the current distribution of MNCH service utilization. Many midwives in CHS could be relocated to DH to align the increased amount of delivery service needed to reduce overwhelmed workload for the DH staff rather than redundant night shift of CHS midwives for the very rare event of delivery there. This policy option further decreases any further investment for too rare delivery services in CHS. Merging the delivery function of several CHS into only one or two CHS located for the remote area from DH can be only sustained while the rest of CHS discontinue the delivery function. The second policy option is an active rearrangement of pregnant mothers’ service utilization pattern, which was more DH and PC oriented to CHS oriented one by a new incentive mechanism and active investment of CHS including ultrasonography and staff refreshment training. This reform can be coupled with merging of some adjacent CHSs into one birthing center for both ANC and delivery services provided to increase the economy of scale per CHS. Either one or both policy options could be applied to improve macro-efficiency so that health system sustainability even under the increasing service load normally coming from successful proceeding of universal health coverage achievement (people’s reducing payment at the point of service utilization) over time. Additionally, the government’s efforts to lower economic barriers to MNCH services through increasing coverage of health insurance are also needed, especially for the vulnerable. Especially in urbanizing cities, inequalities in MNCH service may be widening. Government policies such as free health insurance cards may enable households to access MNCH services of better quality. Further study is needed to determine whether this finding is similar in another rapidly urbanizing rural area in Vietnam.

## Supplementary information

**Additional file 1:** Interview guide questions.

## Data Availability

Not applicable (we do not use any form of dataset).

## Abbreviations

ANC: Antenatal care
CHS: Commune health station
COREQ: Consolidated Criteria for Reporting Qualitative Research
C-section: Caesarian section
DH: District hospital
DHC: District health center
DHO: District health office
DPC: District population center
FGI: Focus group interview
IDI: In-depth interview
MDG: Millennium development goal
MNCH: Maternal, neonatal, and child health
PC: Private clinic
PNC: Postnatal care
