# Supplementary material for: Maternal, neonatal, and child health systems under rapid urbanization: a qualitative study in a suburban district in Vietnam
Source: BMC Health Serv Res. 2020 Feb 5;20:90. doi: 10.1186/s12913-019-4874-7 (PMC7003413; doi:10.1186/s12913-019-4874-7)
Supplement: Supplementary file 1 — Additional file 1: Interview guide questions. [file 12913_2019_4874_MOESM1_ESM.docx]

**Appendix 1. Interview guide questions**

**I. Service providers**

1. **Individual health manager**
2. Please describe the health service delivery system at Quoc Oai district.
3. Please elaborate on the delivery of maternal and child health care.
4. What are the major maternal and child health issues in the district?
5. What are the on-going projects/interventions on maternal and child health being implemented in the district? (both government and donors’ projects/interventions)
6. How do you evaluate these projects/interventions? How effective are they? How helpful are they for the community? What are the changes in the behavior of service providers and service users? In your opinion, what should be done to have these projects/interventions more effective?
7. In your opinion, what interventions should be made immediately and in the longer run to improve the situation of maternal and child health in the district?

**Additional for hospital/health center manager**

7. Please name the maternal and child health services in this health facility.

8. Do you think these number of services are enough? What services should be added up or reduced?

9. Does this health facility have enough equipment, human resources, financial resources, and other resources to implement these services?

10. In your opinion, what should be changed to improve the quality of the services?

1. **Individual health worker (physician, nurse, midwife)**
2. Please name the maternal and child health services in this health facility.
3. Do you think these number of services are enough? What services should be added up or reduced?
4. Does this health facility have enough resources to implement these services at good quality?
5. Please describe your daily work?
6. If there is a new project/intervention implemented in this health facility, how would that change your daily work? Are you willing to have more work from the new project/intervention, please tell why?
7. How do you get paid? (from this health facility? from elsewhere?)
8. Who are your main patients?
9. When do they usually visit you? (when they are very sick or lightly sick)
10. Please describe your difficulties when treating patients? (if any)
11. Are you confident with your knowledge and skills when treating patients?
12. Have you ever received any training to improve your skills?
13. Do you think that you need further training? on what skills to you need?
14. What are the main issues of maternal and child in this community? (issues from patients and issues from service delivery)
15. In your opinion, what knowledge should a pregnant know in order to ensure maternal and child health?
16. How about the knowledge of other people in the family and the community concerning pregnancy, delivery, child health? Do they have enough knowledge? How traditional belief affects their behavior?
17. In your opinion, what habits/behaviors of pregnant women/mothers should be changed to improve maternal and child health?

1. **Group health workers (physician, nurse, midwife)**
2. Please describe your daily work?
3. Who are your main patients? What symptom do they usually have?
4. Please describe your difficulties when treating patients? (if any)
5. Are you confident with your knowledge and skills when treating patients? Do you think that you need further training? on what skills to you need?
6. What are the main issues of maternal and child in this community? (issues from patients and issues from service delivery)
7. In your opinion, what knowledge should a pregnant know in order to ensure maternal and child health?
8. What are the traditional beliefs on maternal health? What is your opinion concerning those beliefs?
9. In your opinion, what habits/behaviors of pregnant women/mothers should be changed to improve maternal and child health?

**II. Service users**

**A. health facilities**

1. **General information**
2. How old are you?/When were you born?
3. How many children do you have?
4. Who do you live with? (family members)
5. Where do you live? Who brought you here today? By what means of transportation? How long did it take? Do you have any difficulties getting here?
6. Is there any health facilities nearby your house? How long does it take to get to the nearest public/private health facility from your house?
7. What is your job? What are the main sources of income for your family?
8. Do you/your family have health insurance? If not, do you have intension to buy health insurance? Are you interested in the national health insurance or private health insurance?

**2. Health seeking behavior**

1. Why you are here today?
2. Who advised you to come here? Why do you choose this health facility?
3. How were you treated today? Please describe in detail.
4. How do you satisfy with the services you received in this health facility?
5. Do you have any difficulties accessing to the services? (financial, lack of family mental support, attitude of health staff etc.)
6. How much do pay for the services you received today? Do you know what costs you paid for? Is this amount of money higher than your expectation? Do you think that health insurance reduces the cost significantly (only the person has health insurance)?
7. Will you continue to seek care at this health facility? Please tell why?
8. Describe the best experience you have here?
9. Describe the worst experience you have here?

**3. Maternal health**

1. Do/Did you come here for check-up during pregnancy?
2. In your opinion, how many times should a pregnant woman need checking up during pregnancy? How many times did you have the check-up? ***(If the numbers are different, ask her “why”?)***
3. In your opinion, where should the pregnant woman deliver? and why?
4. What do you think about postnatal check-up?
5. Do/Did you deliver here? Do you plan to deliver here (for pregnant woman)? And why?
6. How do you evaluate the quality of services of this health facility?
7. What are the main reasons that you chose this health facility?
8. How do you satisfy with the services of this health facility?
9. What do your family members think about the maternal health services: antenatal care, delivery, C-section, postnatal care etc.? Do they support you having these services? What are their reasons?
10. What are the most difficulties that you have when accessing to maternal services? (antenatal, delivery, postnatal)
11. What is your best experience when using these services?
12. What are the costs that you pay for antenatal care? delivery? postnatal care? and today?
13. Do you have to borrow money from family and/or friends to pay for these costs? How does health insurance help?

**4. Child health**

1. How was your baby when he/she was born?
2. How was the baby fed?
3. Up to when did/do you stop/intend to stop breast feeding?
4. When do you bring your child to see a doctor?
5. What kind of symptoms/abnormality that you treat your child at home?

**5. Immunization**

1. Does your child have immunization card?
2. How often does your child get immunized? Who reminds you of the immunization schedule? Do you actively follow the immunization schedule written in the immunization card?
3. After being immunized, does your child usually have abnormal symptoms? Were you informed by health officers about these symptoms? What are the attitude/reactions of your family toward these symptoms? Will you continue having your child immunized?
4. Please tell me why a child need immunizing? How do you know all about the information?
5. How do you satisfy with the quality of the services at this health facility?

**B. At households**

1. **Mother groups**

**a.** **General information**

1. How old are you?/When were you born?
2. How many children do you have? When was the last time you delivered a baby?
3. Who do you live with? (family members)
4. Are there any health facilities nearby your house? How long does it take to get to the nearest public/private health facility from your house?
5. What is your job? What are the main sources of income for your family?
6. Do you/your family have health insurance? If not, do you have intension to buy health insurance? Are you interested in the national health insurance or private health insurance?

**b. Maternal health**

1. When was the last time you were pregnant?
2. Did you visit health facility for pregnant check-up? Which health facility? How many times? Who brought you? Were you encouraged by your family members (husbands, mother in law)?
3. Where did you deliver? Why did you choose that place? What do your family members think about that place?
4. Who assisted you during labor?
5. How was your delivery?
6. How was the attitude of the health staffs?
7. What do you think about the quality of the services? Would you recommend others to go there?
8. How about check-up after delivery?
9. What changes after your delivery concerning eating habit? work burden? rest time?
10. What are the most difficulties accessing maternal services: antenatal? delivery? postnatal?

**c. Child health**

1. How was your baby when he/she was born?
2. How was the baby fed?
3. Up to when did you stop/intend to stop breast feeding?
4. When do you bring your child to see a doctor?
5. What kind of symptoms/abnormality that you treat your child at home?

**d. Immunization**

1. Does your child have immunization card?
2. How often does your child get immunized? Who reminds you of the immunization schedule? Do you actively follow the immunization schedule written in the immunization card?
3. After being immunized, does your child usually have abnormal symptoms? Were you informed by health officers about these symptoms? What are the attitude/reactions of your family toward these symptoms? Will you continue having your child immunized?
4. Please tell me why a child need immunizing? How do you know all about the information?
5. How do you satisfy with the quality of the services at this health facility?
6. What are your family members (husband, the elderly) think about immunization?

**2. The elderly groups**

**a.** **General information**

1. How old are you?/When were you born?
2. How many children do you have?
3. Who do you live with? (family members)
4. Is there any health facilities nearby your house? How long does it take to get to the nearest public/private health facility from your house?
5. When you are sick where do you sick care?
6. What is your job? What are the main sources of income for your family?
7. Do you/your family have health insurance? If not, do you have intension to buy health insurance? Are you interested in the national health insurance or private health insurance?

**b. Maternal health**

1. When was the last time your daughter/daughter-in-law pregnant?
2. What do you think about pregnancy check-up? (does she need it? where should she go? how many time?)
3. In your opinion, what should a pregnant woman be careful of? (food, medicine etc.)
4. Did she take your advice?
5. Did she have any problems during her pregnancy?
6. Where did your daughter/daughter-in-law deliver? Who helped her?
7. After delivery, did your daughter/daughter-in-law and the baby have medical check-up? Do you think that it is necessary?
8. Who decided where the baby should be born? How was your opinion evaluated by other family members? Do you believe in your own experience or what is informed by health workers?
9. What is your opinion about the place of delivery? Where is the best place?
10. What do you think about food in-take after delivery? work burden after delivery? Please share the experience of your daughter/daughter-in-law concerning this issue
11. What are the most difficulties in accessing to maternal care?

**c. Child health**

1. How was your grandchild when he/she was born?
2. How was your grandchild fed?
3. In your opinion how long should a baby be breast fed?

**d. Immunization**

1. Does your grandchild have immunization card?
2. How often does your grandchild get immunized?
3. What do you think about immunization?
4. Please tell me why a child need immunizing? How do you know all about the information?
5. Do you encourage your grandchild be immunized?

**3. Individual mothers**

**a.** **General information**

1. How old are you?/When were you born?
2. How many children do you have?
3. Who do you live with? (family members)
4. Are there any health facilities nearby your house? How long does it take to get to the nearest public/private health facility from your house?
5. What is your job? What are the main sources of income for your family?
6. Do you/your family have health insurance? If not, do you have intension to buy health insurance? Are you interested in the national health insurance or private health insurance?

**b. Maternal health**

1. When was the last time you delivered a baby?
2. Have you ever had an experience of unwanted/unplanned pregnancy? What did you do in that circumstance?
3. Did you visit health facility for pregnant check-up? Which health facility? How many times? Who brought you? Were you encouraged by your family members (husbands, mother in law)?
4. Did you have any problem during pregnancy? (If yes, interviewer further explores on the problem and how the problem was solved)
5. Do you have any food restriction during pregnancy? What are they?
6. Where did you deliver? Why did you choose that place? What do your family members think about that place?
7. Who assisted you during labor?
8. How was your delivery?
9. How was the attitude of the health staffs?
10. What do you think about the quality of the services? Would you recommend others to go there?
11. How about check-up after delivery?
12. What changes after your delivery concerning eating habit? work burden? rest time?
13. What are the most difficulties accessing maternal services: antenatal? delivery? postnatal?

**c. Child health**

1. How was your baby when he/she was born?
2. How was the baby fed?
3. Up to when did you stop/intend to stop breast feeding?
4. When do you bring your child to see a doctor?
5. What kind of symptoms/abnormality that you treat your child at home?

**d. Immunization**

1. Does your child have immunization card?
2. How often does your child get immunized? Who reminds you of the immunization schedule? Do you actively follow the immunization schedule written in the immunization card?
3. After being immunized, does your child usually have abnormal symptoms? Were you informed by health officers about these symptoms? What is the attitude/reactions of your family toward these symptoms? Will you continue having your child immunized?
4. Please tell me why a child need immunizing? How do you know all about the information?
5. How do you satisfy with the quality of the services at this health facility?
6. What are your family members (husband, the elderly) think about immunization?

**4. The elderly individual**

**a.** **General information**

1. How old are you?/When were you born?
2. How many children do you have?
3. Who do you live with? (family members)
4. Are there any health facilities nearby your house? How long does it take to get to the nearest public/private health facility from your house?
5. When you are sick where do you seek care?
6. What is your job? What are the main sources of income for your family?
7. Do you/your family have health insurance? If not, do you have intension to buy health insurance? Are you interested in the national health insurance or private health insurance?

**b. Maternal health**

1. When was the last time your daughter/daughter-in-law pregnant?
2. What do you think about pregnancy check-up? (does she need it? where should she go? how many times?)
3. In your opinion, what should a pregnant woman be careful of? (food, medicine etc.)
4. Did she take your advice?
5. Did she have any problems during her pregnancy?
6. Where did your daughter/daughter-in-law deliver? Who helped her?
7. After delivery, did your daughter/daughter-in-law and the baby have medical check-up? Do you think that it is necessary?
8. Who decided where the baby should be born? How was your opinion evaluated by other family members? Do you believe in your own experience or what is informed by health workers?
9. What is your opinion about the place of delivery? Where is the best place?
10. Should your daughter/daughter-in-law have the same experience with other women in the village concerning pregnancy, delivery, and postnatal care?
11. What do you think about food in-take after delivery? work burden after delivery? Please share the experience of your daughter/daughter-in-law concerning this issue
12. What are the most difficulties in accessing to maternal care?
13. Do you want your daughter/daughter-in-law to have more children? why?

**c. Child health**

1. How was your grandchild when he/she was born?
2. How was your grandchild fed?
3. In your opinion how long should a baby be breast fed?

**d. Immunization**

1. Does your grandchild have immunization card?
2. How often does your grandchild get immunized?
3. What do you think about immunization?
4. Please tell me why a child need immunizing? How do you know all about the information?
5. Do you encourage your grandchild be immunized?
